# Frequency of pathogenic germline variants in cancer susceptibility genes in 1336 renal cell carcinoma cases

**DOI:** 10.1093/hmg/ddac089

**Published:** 2022-04-20

**Authors:** Bryndis Yngvadottir, Avgi Andreou, Laia Bassaganyas, Alexey Larionov, Alex J Cornish, Daniel Chubb, Charlie N Saunders, Philip S Smith, Huairen Zhang, Yasemin Cole, Genomics England Research Consortium, James Larkin, Lisa Browning, Samra Turajlic, Kevin Litchfield, Richard S Houlston, Eamonn R Maher

**Affiliations:** Department of Medical Genetics, School of Clinical Medicine, University of Cambridge, Cambridge, CB2 0QQ, UK; Department of Medical Genetics, School of Clinical Medicine, University of Cambridge, Cambridge, CB2 0QQ, UK; Department of Medical Genetics, School of Clinical Medicine, University of Cambridge, Cambridge, CB2 0QQ, UK; Department of Medical Genetics, School of Clinical Medicine, University of Cambridge, Cambridge, CB2 0QQ, UK; School of Water, Energy and Environment, Cranfield University, Cranfield, MK43 0AL, UK; Division of Genetics and Epidemiology, The Institute of Cancer Research, London, SW7 3RP, UK; Division of Genetics and Epidemiology, The Institute of Cancer Research, London, SW7 3RP, UK; Division of Genetics and Epidemiology, The Institute of Cancer Research, London, SW7 3RP, UK; Department of Medical Genetics, School of Clinical Medicine, University of Cambridge, Cambridge, CB2 0QQ, UK; Department of Medical Genetics, School of Clinical Medicine, University of Cambridge, Cambridge, CB2 0QQ, UK; Department of Medical Genetics, School of Clinical Medicine, University of Cambridge, Cambridge, CB2 0QQ, UK; Genomics England, Queen Mary University of London, Dawson Hall, Charterhouse Square, London, EC1M 6BQ, UK; William Harvey Research Institute, Queen Mary University of London, London, EC1M 6BQ, UK; Department of Medical Oncology, Renal and Skin Units, The Royal Marsden NHS Foundation Trust, London, SW3 6JJ, UK; Melanoma and Kidney Cancer Team, The Institute of Cancer Research, London, SW7 3RP, UK; Department of Cellular Pathology, Oxford University Hospitals NHS Foundation Trust, John Radcliffe Hospital, Oxford, OX3 9DU, UK; NIHR Oxford Biomedical Research Centre, Oxford University Hospitals NHS Foundation Trust, Oxford, OX4 2PG, UK; Department of Medical Oncology, Renal and Skin Units, The Royal Marsden NHS Foundation Trust, London, SW3 6JJ, UK; Melanoma and Kidney Cancer Team, The Institute of Cancer Research, London, SW7 3RP, UK; Cancer Dynamics Laboratory, The Francis Crick Institute, London, NW1 1AT, UK; Department of Oncology, University College London Cancer Institute, Paul O’Gorman Building, London, WC1E 6DD, UK; Division of Genetics and Epidemiology, The Institute of Cancer Research, London, SW7 3RP, UK; Department of Medical Genetics, School of Clinical Medicine, University of Cambridge, Cambridge, CB2 0QQ, UK

## Abstract

Renal cell carcinoma (RCC) occurs in a number of cancer predisposition syndromes, but the genetic architecture of susceptibility to RCC is not well defined. We investigated the frequency of pathogenic and likely pathogenic (P/LP) germline variants in cancer susceptibility genes (CSGs) within a large series of unselected RCC participants. Whole-genome sequencing data on 1336 RCC participants and 5834 controls recruited to the UK 100 000 Genomes Project, a nationwide multicentre study, was analyzed to identify rare P/LP short variants (single nucleotide variants and insertions/deletions ranging from 1 to 50 base pairs) and structural variants in 121 CSGs. Among 1336 RCC participants [mean: 61.3 years (±12 SD), range: 13–88 years; 64% male], 85 participants [6.4%; 95% CI (5.1, 7.8)] had one or more P/LP germline variant in a wider range of CSGs than previously recognized. A further 64 intragenic variants in CSGs previously associated with RCC were classified as a variant of uncertain significance (VUS) (24 ‘hot VUSs’) and were considered to be of potential clinical relevance as further evaluation might results in their reclassification. Most patients with P variants in well-established CSGs known to predispose to renal cell carcinoma (RCC-CSGs) were aged <50 years. Burden test analysis for filtered variants in CSGs demonstrated a significant excess of *CHEK2* variants in European RCC participants compared with the healthy European controls (*P* = 0.0019). Approximately, 6% of the patients with RCC unselected for family history have a germline variant requiring additional follow-up analysis. To improve diagnostic yield, we suggest expanding the panel of RCC-CSGs tested to include *CHEK2* and all *SDH*x subunits and raising the eligibility criteria for age-based testing.

## Introduction

Kidney cancer is the sixth most commonly diagnosed cancer in the more developed regions of the world and the incidence rates have been rising (1,2). Renal cell carcinoma (RCC) comprises over 90% of kidney cancers and clear cell renal cell carcinoma (ccRCC) is the major histological subtype (∼75% of patients), with papillary RCC (pRCC types 1 and type 2), chromophobe RCC (chRCC) and rarer forms accounting for the remainder of patients (15, 5, 5%) (3,4).

Risk factors for kidney cancer include obesity, smoking, hypertension and multiple autosomal dominantly inherited cancer predisposition syndromes including von Hippel–Lindau (VHL), Birt-Hogg-Dubé (BHD) syndrome, hereditary leiomyomatosis and renal cell cancer syndrome (HLRCC), *PTEN* hamartoma tumour syndrome, hereditary pRCC, *BAP1* tumour predisposition syndrome, succinate dehydrogenase subunit genes (*SDHB*, *SDHC* and *SDHD*) and constitutional chromosome 3 translocations (2,5,6). Common single-nucleotide polymorphisms also influence RCC risk, affirming a complex heritable basis, but one that is likely to be shaped predominantly by rare variants (7,8).

Although only 3% of RCC patients have a family history of the disease, germline pathogenic variants in cancer susceptibility genes (CSGs) have been reported to be detectable in up to 16% of a referral-based cohort of advanced RCC (9). The contribution of germline variants reported from different centres varies considerably as a consequence of which genes were tested and there were variations in patient ascertainment and selection (9–12). To provide a comprehensive understanding of the contribution of pathogenic/likely pathogenic (P/LP) variants in 121 CSGs to RCC development, we analyzed the whole-genome sequencing (WGS) data on 1336 individuals with RCC recruited into the UK’s 100 000 Genomes Project (100kGP) (13).

## Results

### Prevalence of P/LP variants in CSGs

**Figure 1:**
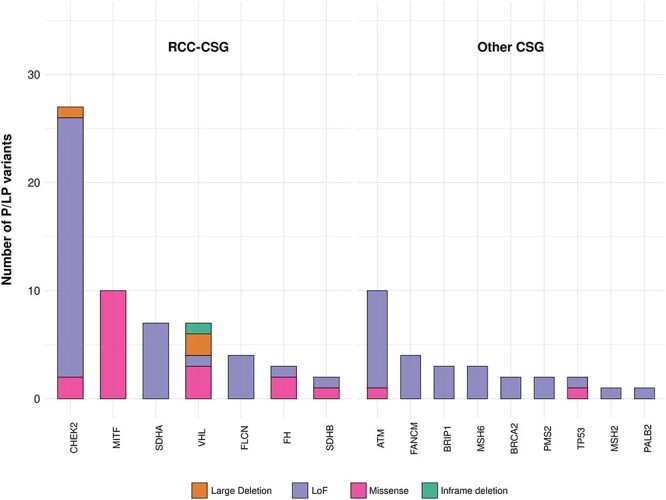
Frequency of P/LP variants in CSGs (RCC-CSGs and other CSGs) in RCC participants. The LoF category includes stop gained, stop lost, frameshifts and splicing variants (individually listed in Supplementary Material, Table S1A).

The CSGs harbouring clinically relevant variants were subdivided into CSGs known to predispose to renal cell carcinoma (RCC-CSGs) and CSGs CSGs (other CSGs) not previously associated with RCC. All P/LP variants (total 88 variants) were heterozygous, 85 short [single nucleotide variants (SNVs) and insertions/deletions ranging from 1 to 50 base pairs (INDELs)] and three structural variants (SVs) (deletions). Around 68.2% (60/88) of 88 P/LP variants detected were in RCC-CSGs and 31.8% (28/88) were in the other CSGs (all autosomal dominant predisposition genes) (Fig. 1, Tables 1 and 2).

**Table 1:** P/LP variants identified in well-established RCC-CSGs in a cohort of 1336 RCC participants

Gene	HGVSc^a^	HGVSp^b^	No. of participants (%)
*CHEK2*	ENST00000382580.6:c.1229del	ENSP00000372023.2:p.Thr410MetfsTer15	16 (1.2)
*CHEK2*	ENST00000382580.6:c.1392del	ENSP00000372023.2:p.Ser465ValfsTer15	4 (0.3)
*CHEK2*	ENST00000382580.6:c.478A>G	ENSP00000372023.2:p.Arg160Gly	2 (0.1)
*CHEK2*	ENST00000382580.6:c.573 + 1G>A		2 (0.1)
*CHEK2*	ENST00000382580.6:c.1031del	ENSP00000372023.2:p.Leu344TrpfsTer3	1 (0.07)
*CHEK2*	ENST00000382580.6:c.720del	ENSP00000372023.2:p.Val241PhefsTer7	1 (0.07)
*CHEK2*	7.5 kb del		1 (0.07)
*MITF*	ENST00000448226.7:c.1273G>A	ENSP00000391803.2:p.Glu318Lys	10 (0.7)
*SDHA*	ENST00000264932.11:c.91C>T	ENSP00000264932.6:p.Arg31Ter	7 (0.5)
*VHL*	ENST00000256474.2:c.227_229del	ENSP00000256474.2:p.Phe76del	1 (0.07)
*VHL*	ENST00000256474.2:c.233A>G	ENSP00000256474.2:p.Asn78Ser	1 (0.07)
*VHL*	ENST00000256474.2:c.461C>T	ENSP00000256474.2:p.Pro154Leu	1 (0.07)
*VHL*	ENST00000256474.2:c.286C>T	ENSP00000256474.2:p.Gln96Ter	1 (0.07)
*VHL*	ENST00000256474.2:c.551T>C	ENSP00000256474.2:p.Leu184Pro	1 (0.07)
*VHL*	13 kb del		1 (0.07)
*VHL*	10 kb del		1 (0.07)
*FLCN*	ENST00000285071.9:c.33C>A	ENSP00000285071.4:p.Cys11Ter	1 (0.07)
*FLCN*	ENST00000285071.9:c.490del	ENSP00000285071.4:p.Arg164GlyfsTer13	1 (0.07)
*FLCN*	ENST00000285071.9:c.890_893del	ENSP00000285071.4:p.Glu297AlafsTer25	1 (0.07)
*FLCN*	ENST00000285071.9:c.853C>T	ENSP00000285071.4:p.Gln285Ter	1 (0.07)
*FH*	ENST00000366560.3:c.1127A>C	ENSP00000355518.3:p.Gln376Pro	1 (0.07)
*FH*	ENST00000366560.3:c.431G>T	ENSP00000355518.3:p.Gly144Val	1 (0.07)
*FH*	ENST00000366560.3:c.413_414del	ENSP00000355518.3:p.Leu138ArgfsTer17	1 (0.07)
*SDHB*	ENST00000375499.7:c.72 + 1G>T		1 (0.07)
*SDHB*	ENST00000375499.7:c.600G>T	ENSP00000364649.3:p.Trp200Cys	1 (0.07)

^a^HGVSc: Human Genome Variation Society coding sequence name.

^b^HGVSp: Human Genome Variation Society protein sequence name.

The genotype and phenotypes of the RCC participants with rare P/LP germline variants are summarized in Supplementary Material, Table S1A. Two cases of multilocus inherited neoplasia alleles syndrome (14) were detected: a male with *CHEK2* and *ATM* variants and another male with a *CHEK2* and two *MSH6* P/LP variants, both with ccRCC (Supplementary Material, Table S1A).

The highest prevalence of P/LP germline variants in RCC-CSGs was in *CHEK2*, with 27 individuals (seven unique variants) harbouring P/LP variants within the gene [27/1336 (2%); 24 of these were loss of function (LoF) variants]. Other genes from this group with germline variants included *MITF* [10/1336 (0.7%)], *SDHA* [7/1336 (0.5%)], *VHL* [7/1336 (0.5%)], *FLCN* [4/1336 (0.3%)], *FH* [3/1336 (0.2%)] and *SDHB* [2/1336 (0.1%)].

The highest number of P/LP germline variants in the other CSG group was in *ATM*, with 10 individuals (9 unique variants) harbouring P/LP variants [10/1336 (0.7%)]. In addition, P/LP germline variants were detected in *FANCM* [4/1336 (0.3%)], *BRIP1* [3/1336 (0.2%)], *MSH6* [3/1336 (0.2%)], *BRCA2* [2/1336 (0.1%)], *PMS2* [2/1336 (0.1%)], *TP53* (2/1336 (0.1%)], *MSH2* [1/1336 (0.07%)] and *PALB2* [1/1336 (0.07%)]. The individual variants are summarized in Table 2.

A further 64 variants in RCC-CSGs were classified as a variant of uncertain significance (VUS) but were considered to be of potential clinical relevance as further evaluation (e.g. by detailed clinical genetic assessment, tumour immunohistochemistry or family studies) might result in the reclassification of these variants as P/LP (Supplementary Material, Table S1B). No VUSs in other CSGs were considered as clinically relevant in the context of RCC. In order to clarify the 10–90% range of potential pathogenicity for the 54 SNV VUSs, we used the quantitative Bayesian framework provided by Tavtigian *et al*. (15) to calculate a posterior probability and then classified them to hot/warm/tepid or cool/cold/ice cold VUS according to the Association for Clinical Genomic Science (ACGS) guidelines. In summary, there were 24 ‘hot’, 6 ‘warm’, 15 ‘tepid’ and 9 ‘cool/cold’ SNV VUSs. For the remaining 10 CNV VUSs, we used the CNV score based on the American College of Medical Genetics and Genomics (ACMG)/Clinical Genome Resource CNV loss and gain guidelines (2020) (16) (Supplementary Material, Table S1B).

### Candidate rare SVs

**Figure 2:**
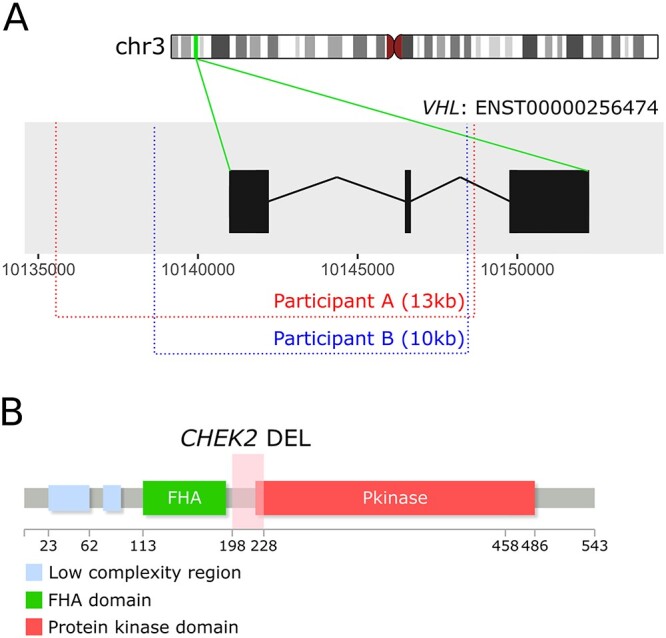
Germline deletions identified in RCC-CSGs. (**A**) Two *VHL* gene exons are deleted in two participants. Participant A has a 13 kb deletion (chr3:10 135 484–10 148 568, GRCh38) and the breakpoint locations are displayed in red on the MANE select *VHL* transcript (ENST00000256474). Participant B has a 10 kb deletion (chr3:10 138 433–10 148 506, GRCh38) with the breakpoints shown in blue (created with Bioconductor’s ggbio package (56), with some adaptation). (**B**) A 7.5 kb deletion involving *CHEK2* was identified in one participant. The deletion (pink shaded area) takes out part of the protein kinase domain (created with (57)).

Seventy-four candidate rare germline SVs (41 deletions, 13 duplications, 14 inversions and 6 translocations) with at least one breakpoint overlapping 31 CSGs (8 RCC-CSGs and 34 other CSGs) were identified in 6.9% (86/1254) participants (Supplementary Results, Supplementary Material, Fig. S1, Supplementary Material, Table S2). We focused on deletions in RCC-CSGs, and three deletions (two in *VHL* and one in *CHEK2*) were considered to be pathogenic (included in the prevalence of P/LP before) without additional functional validation. One of the participants had a 13 kb deletion starting 6 kb upstream of *VHL* in the non-coding sequence, removing 5 kb of the gene, including two of the three exons (Participant A, Fig. 2A). This participant had clinical evidence of VHL disease and did not carry any other P/LP variants. Further analysis, using less stringent filtering (see Supplementary Methods), identified a second germline *VHL* deletion in another participant with a typical VHL phenotype (Participant B, Fig. 2A). A 7.5 kb deletion in *CHEK2*, which removes the fifth exon of the gene and deletes a part of the protein kinase domain (Fig. 2B), was detected in a participant with later onset ccRCC, with the initial filters applied.

**Table 2:** P/LP germline variants identified in other CSGs in a cohort of 1336 RCC participants

Gene	HGVSc^a^	HGVSp^b^	No. of participants (%)
*ATM*	ENST00000278616.8:c.1339C>T	ENSP00000278616.4:p.Arg447Ter	2 (0.1)
*ATM*	ENST00000278616.8:c.964_968del	ENSP00000278616.4:p.Glu322LysfsTer6	1 (0.07)
*ATM*	ENST00000278616.8:c.1442T>G	ENSP00000278616.4:p.Leu481Ter	1 (0.07)
*ATM*	ENST00000278616.8:c.1782del	ENSP00000278616.4:p.Val595CysfsTer19	1 (0.07)
*ATM*	ENST00000278616.8:c.2466 + 1G>A		1 (0.07)
*ATM*	ENST00000278616.8:c.3451A>T	ENSP00000278616.4:p.Lys1151Ter	1 (0.07)
*ATM*	ENST00000278616.8:c.8147T>C	ENSP00000278616.4:p.Val2716Ala	1 (0.07)
*ATM*	ENST00000278616.8:c.652C>T	ENSP00000278616.4:p.Gln218Ter	1 (0.07)
*ATM*	ENST00000278616.8:c.742C>T	ENSP00000278616.4:p.Arg248Ter	1 (0.07)
*FANCM*	ENST00000267430.10:c.5101C>T	ENSP00000267430.5:p.Gln1701Ter	2 (0.07)
*FANCM*	ENST00000267430.10:c.1972C>T	ENSP00000267430.5:p.Arg658Ter	1 (0.07)
*FANCM*	ENST00000267430.10:c.3235_3238del	ENSP00000267430.5:p.Leu1080ValfsTer14	1 (0.07)
*BRIP1*	ENST00000259008.6:c.3401del	ENSP00000259008.2:p.Pro1134LeufsTer16	1 (0.07)
*BRIP1*	ENST00000259008.6:c.2992_2995del	ENSP00000259008.2:p.Lys998GlufsTer60	1 (0.07)
*BRIP1*	ENST00000259008.6:c.2392C>T	ENSP00000259008.2:p.Arg798Ter	1 (0.07)
*MSH6*	ENST00000234420.9:c.3261del	ENSP00000234420.4:p.Phe1088SerfsTer2	1 (0.07)
*MSH6*	ENST00000234420.9:c.3259_3260insT	ENSP00000234420.4:p.Pro1087LeufsTer6	1 (0.07)
*MSH6*	ENST00000234420.9:c.3562_3563del	ENSP00000234420.4:p.Ser1188TyrfsTer5	1 (0.07)
*BRCA2*	ENST00000380152.7:c.9253dup	ENSP00000369497.3:p.Thr3085AsnfsTer26	1 (0.07)
*BRCA2*	ENST00000380152.7:c.4876_4877del	ENSP00000369497.3:p.Asn1626SerfsTer12	1 (0.07)
*PMS2*	ENST00000265849.12:c.1A>G	ENSP00000265849.7:p.Met1?	1 (0.07)
*PMS2*	ENST00000265849.12:c.1778del	ENSP00000265849.7:p.Lys593SerfsTer2	1 (0.07)
*TP53*	ENST00000269305.8:c.655C>T	ENSP00000269305.4:p.Pro219Ser	1 (0.07)
*TP53*	ENST00000269305.8:c.586C>T	ENSP00000269305.4:p.Arg196Ter	1 (0.07)
*MSH2*	ENST00000233146.6:c.942_942+2del	ENSP00000233146.2:p.Val265_Gln314del	1 (0.07)
*PALB2*	ENST00000261584.8:c.3113G>A	ENSP00000261584.4:p.Trp1038Ter	1 (0.07)

^a^HGVSc: Human Genome Variation Society coding sequence name.

^b^HGVSp: Human Genome Variation Society protein sequence name.

Combining the results for intragenic and copy number variant analysis, an overall diagnostic yield of 6.4% [95% CI (5.1, 7.8)] was calculated (82/1336 participants with a germline P/LP short variant and 3/1254 with a P/LP SV).

### Genotype–phenotype relationship

#### Additional non-RCC tumours

Four of the RCC participants with germline *VHL* P/LP variants had clinical features characteristic of VHL disease (haemangioblastomas, multiple ccRCCs and spinal cord tumours). In contrast, none of the 7 participants with *FH* or *FLCN* mutations were reported to have clinical indicators of HLRCC or BHD syndrome and none of the 10 carriers of P *MITF* variants had a past history of melanoma. Although 10 of 60 participants with a P/LP variant in an RCC-CSG had an additional non-RCC neoplasm (breast cancer, colorectal cancer, thyroid cancer, ovarian cancer, testicular tumour, basal cell carcinoma and haematological malignancy), none of the tumour combinations were characteristic for a recognized inherited RCC syndrome.

Seven of 28 participants with a P/LP variant in other CSGs had a past history of non-RCC cancer (bladder cancer, prostate cancer, testicular cancer and breast cancer), including a case with a germline *TP53* mutation with synchronous uterine cancer, central nervous system cancer and chRCC at 45 years. Breast cancer was recorded in one of ten participants with a P/LP *ATM* variant. None of the participants with mismatch repair (MMR) or *POLE* P/LP variants had a history of colorectal cancer and their tumours did not show a cancer MMR signature.

#### Gender

There was no significant difference between the frequency of P/LP variants in males (5.7% (49/854)) and females [7.5% (36/482)] (*P* = 0.24).

#### Age

**Figure 3:**
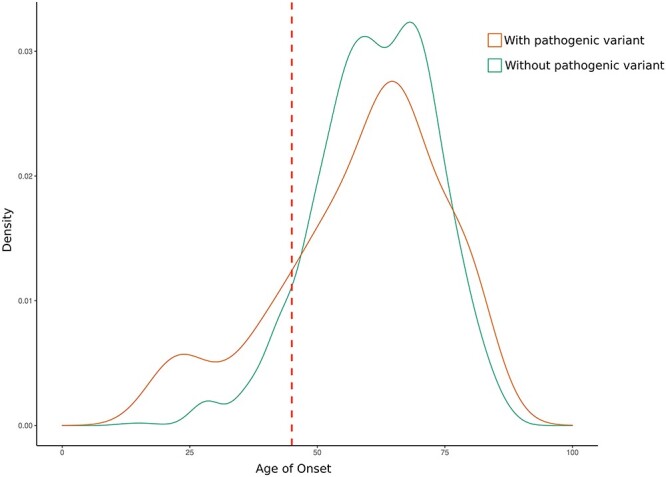
Onset of RCC in participants with and without a P/LP variant. Dotted red line shows the age cut-off (46 years) for offering germline testing.

RCC participants with a P/LP variant tended to be younger (mean: 58.6 years versus 61.5 years; *P* = 0.10; Fig. 3). This was also observed for RCC participants with a P/LP in an RCC-CSG compared with other CSGs (mean: 58.0 years versus 59.9 years; *P* = 0.55; Supplementary Material, Table S3). Of the 19 early onset (≤45 years) participants with a P/LP variant in an RCC-CSG, the majority were in *VHL* (*n* = 7), followed by *CHEK2* (*n* = 3), *FLCN* (*n* = 2) and *SDHB* (*n* = 2). Mean age of RCC onset in individuals with *VHL* and *CHEK2* P/LP variants was 25.6 years (range: 18–40 years) and 64.7 years (range: 39–84 years), respectively. Of the five early onset (≤45 years) participants with a P/LP variant in other CSGs, the genes involved were *ATM* (*n* = 2), *BRIP1* (*n* = 1), *TP53* (*n* = 1) and *PALB2* (*n* = 1). Applying an age cut-off of <46 years would have detected a P/LP variant in 1.3% (18/1336) of the entire cohort and would have identified only 23.3% (14/60) of participants with a P/LP variant in an RCC-CSG (Supplementary Material, Table S4).

#### Histology

Participants with non-ccRCCs were more commonly associated with germline P/LP variants than clear cell [8.5% (19/224) and 5.8% (53/912), respectively], but the difference was not statistically significant (*P* = 0.17). Of the 72 participants with P/LP germline variants and detailed histology available, 73.6% (53/72) were classified as ccRCC and 26.4% (19/72) had a non-clear tumour. For full information on histopathology, see Table 3 and Supplementary Material, Table S1A and B; but in brief, germline *VHL* variants were associated with ccRCC (*n* = 4 ccRCC, *n* = 3 histology not available), two chRCCs were seen in association with a germline *FLCN* variant (overall histologies in four participants with a P/LP *FLCN* variants: *n* = 2 chRCC, *n* = 1 oncocytic, *n* = 1 histology not available) and *FH* variants were seen in three participants, whose RCC tumour was classified as collecting duct (*n* = 1), ccRCC (*n* = 1) and the histology of the remaining one was not available.

**Table 3:** Characteristics of 1336 participants with RCC

Participants with RCC		*N* = 1336	%
Age (years)	Mean (range)	61.3 (13–88)	
Sex			
Male		854	63.9
Female		482	36.1
Ethnicity (PCA-based)			
Predominantly European ancestries		1184	89
Predominantly South and East Asian ancestries		53	4
Predominantly African ancestries		27	2
Other		72	5
Ethnicity (self-reported)			
White British		937	70.1
Other White background		67	5.0
Asian (Indian, Pakistani, Bangladeshi and other Asian)		38	2.8
Black (Caribbean, African and other Black)		20	1.5
Mixed background		7	0.5
Other ethnic group		20	1.5
Not stated		175	13.1
Not available		72	5.4
Number of RCC tumours			
1		1287	76.3
≥2		48	3.6
Personal history of other cancer			
0		1090	81.6
1		197	14.7
2		36	2.7
≥3		13	1.0
Histology^a^		*N* = 1388^a^	
Clear cell		939	67.7
Non-clear cell		237	17.0
Unspecified RCC histology		149	10.7
Not available		61	4.4
Uncertain malignancy		<5^a^	0.1
Tumour stage		*N* = 1388^a^	
1		553	39.9
2		123	8.9
3		420	30.3
4		116	8.4
Unclassified		<5	0.1
Not available		174	12.5

PCA, Principal component analysis.

^a^Histology and Stage numbers shown are for RCC tumours overall in our cohort, as there were 48 participants with ≥2 RCC tumours.

Most *CHEK2*-associated tumours were classified as ccRCCs [19 ccRCC, five non-ccRCC (*n* = 4 chRCC, *n* = 1 oncocytic) and *n* = 3 histology not available]. For the participants with a *MITF* subunit variant, four were classified as ccRCC, four as non-ccRCC (*n* = 2 chRCC, *n* = 2 papillary) and, for the remaining two, histology was not available. Of those with an *SDH* subunit variant, five were classified as ccRCC, with the remainder classified as pRCC (*n* = 1), chRCC (*n* = 1) and, for two, histology was not available. More specifically, seven patients shared the same variant in *SDHA*, four of which were classified as ccRCC, two as a non-ccRCC (*n* = 1 papillary, *n* = 1 chRCC) and, for one, histology was not available. Of the two participants with a P/LP *SDHB* variant, one had a ccRCC and the histology for the other was not available.

#### Stage

There was no significant difference between the frequency of germline P/LP CSG variants in non-advanced and advanced RCC [6.9% (45/649) versus 5.5% (29/529); (*P* = 0.24)].

### Burden test results

Burden test analysis showed an excess of *CHEK2* variants, that passed our stringent filtering as detailed in Supplementary Methods, in European RCC participants compared with the healthy European controls that reached statistical significance [Fisher’s false discovery rate (FDR) adjusted *P* = 0.0019] (Supplementary Material, Table S5), confirming an association of *CHEK2* with the RCC phenotype. For other CSGs, an excess of variants was seen in European cases compared with controls in the following genes: *ATM, AXIN2, BAP1, BLM, BMPR1A, BRIP1, CBL, CDKN1B, CDKN1C, CDKN1C, CDKN2A, CYLD, DDB2, DIS3L2, ELANE, EPCAM, EZH2, FANCA, FANCD2, FANCE, FANCG, FANCI, FH, HRAS, MAX, MET, MLH1, NBN, NTHL1, PMS1, POLE, POLH, PTCH1, RB1, RECQL4, RHBDF2, SBDS, SDHA, SDHAF2, SDHB, SDHC, SLC25A13, STK11, TP53, VHL, WRN, XPA* and *XPC*, but none of these genes in contrast to *CHEK2* showed a statistically significant association (*P*-values are available in Supplementary Material, Table S5).

## Discussion

The availability of WGS data has allowed us to provide a more comprehensive appraisal of the contribution of germline pathogenic variants (including SNVs, INDELs and SVs) in CSGs to RCC in an unselected patient cohort. Our analysis suggests an overall detection rate of 6.4% and most P/LP variants were detected in CSGs known to predispose to RCC [4.5% (60/1336); 95% CI (3.4, 5.7)]. P/LP variants in other CSGs (*ATM, FANCM, BRIP1, MSH6, BRCA2, PMS2* and *TP53*) found in 2% participants could reflect a background prevalence in the population or an association with RCC that has not yet been validated. To our knowledge, *ATM* has not previously been implicated in RCC, but truncating *BRIP1* variants have been reported in a subset of patients with inherited RCC (17,18). Further studies are required to confirm potential links. A further 4.7% [64/1336; 95% CI (3.7, 6.1)] of participants had a VUS in an RCC-CSG which was considered to be clinically relevant. Further studies into family history and tumour immunohistochemistry and a more detailed clinical assessment would be needed to evaluate the true relevance of these VUSs in order to upgrade their status to P/LP, but 24 were classified as ‘hot’ VUSs (16). Reclassification of these 24 variants to LP would increase the overall diagnostic yield to 8.2%.

Our diagnostic yield of 6.4% is lower than that reported in studies enriched for a young RCC onset and/or later stage disease. Wu *et al.* (10) reported a diagnostic yield of 9.5% in an RCC cohort (*n* = 190) of young patients (<45 years) which had germline testing on a 23-gene panel and a diagnostic yield of 16.1% was reported in an RCC cohort (*n* = 254) enriched for advanced RCC (World Health Organization stage 3/4) referred for germline testing (76 CSGs analyzed) (9). In a recent large referral-based study (*n* = 1829), 10.3% of participants had clinically actionable P/LP variants and there were some interethnic differences of variant frequency in specific genes (*FH* and *CHEK2*) (11). However, our diagnostic yield is comparable with the 6% reported by the Pan-Cancer Atlas study of 742 cases (19) and the 6.1% reported in a referral-based analysis of 1235 RCC patients (30% with family history) using a panel of 19 genes (12). Interstudy variations likely reflect patient ascertainment and selection and the extent of genetic testing, but our results provide a good estimate of diagnostic yield by comprehensive testing in an unselected series.

In the UK, patients with suspected inherited RCC are examined for features of an inherited cancer syndrome and offered gene panel testing that includes *VHL*, *MET*, *FLCN*, *SDHB*, *FH* and *BAP1* (20). Other countries and commercial laboratories often include additional RCC-CSGs such as *TSC1*, *TSC2, PTEN*, *TP53* and *SDHC/D*. In our study, 44 participants had variants in either *CHEK2* (*n* = 27), *MITF* (*n* = 10) or *SDHA* (*n* = 7) which are not routinely tested for in the UK*.* While *CHEK2* variation was originally identified as conferring a 2-fold increase in breast cancer risk (21), it is increasingly being recognized that variants predispose to other cancers, including colorectal (22) and prostate (23), and more recently has been linked to RCC with studies suggesting a lifetime risk of 2% (24–26).

The *MITF* (*E318K/p.Glu419Lys*) variant (rs149617956) was present in 10 participants. This variant was initially linked to RCC in a study of individuals with RCC, and malignant melanoma and functional studies of this variant demonstrated *MITF* upregulation through loss of a SUMOylation site (27,28). Though subsequent studies have confirmed an association with melanoma (29), a recent meta-analysis failed to demonstrate a significant association with RCC (30).

SDH-associated RCC is more commonly associated with *SDHB* mutations with carriers having an estimated 5% lifetime risk of RCC (31,32). However, in our cohort, germline *SDHA* variants were more common than *SDHB* variants. Germline mutations in *SDHx* are a major cause of phaeochromocytoma and paragangliomas but inheritance patterns and risks differ between genes, and the penetrance of germline *SDHA* mutations is much lower than for *SDHB, SDHC* or *SDHD* (33–35).

There are a number of limitations to our study. Although participants were recruited from a large number of individual centres and were not selected for any specific characteristics, all were fit to undergo surgery. In addition, , there was no centralized review of histopathology, some clinical data were not available for all participants and data on the presence of syndromic RCC extra-renal manifestations and family history were not collected.

Our findings have implications for the application of genetic testing for germline variants in individuals with RCC. Though most centres offer testing to patients with features of an inherited cancer syndrome, there is less consensus for testing isolated non-syndromic cases. Within the UK, testing is offered to patients <40 years of age (or <50 years of age for pRCC), but internationally, it has been suggested that an age cut-off <46 years (equivalent to the 10th percentile) would maximize the sensitivity and specificity and an age cut-off of 50 has also been recommended (20,36–39). However, in our cohort, only 23.3% (14/60) of those with an RCC-CSG variant, were aged <46 years. Although there was a trend for a younger age at diagnosis in the genes most frequently tested in clinical practice (*VHL*, *SDHB*, *FH* and *FLCN*; mean: 38 years), mutations in less penetrant genes were detected, on average, in older patients (e.g. *CHEK2* and *SDHA*; mean: 65.5 years). This makes it difficult to define an age cut-off that would efficiently enable the identification of all cases with an RCC-CSG variant without testing the majority of the cohort. In addition, we did not find statistically significant associations in our series with tumour stage or histology (e.g. ccRCC or non-clear cell). In rare cases, characteristic histopathological features may suggest an underlying inherited disorder (e.g. SDH-deficient RCC, hybrid chromophobe-oncocytic and BHD syndrome), but, in general, in the absence of family history or multicentric disease, age at diagnosis seems to be the most practical approach (with 70% general consensus) (38) for stratifying genetic testing. Based on our results, testing a further 106 participants presenting between 45 and 50 years of age would enable detection of an extra 5 participants (Supplementary Material, Table S4**)**.Therefore, we suggest that genetic testing should be extended to <50 years of age and that the small clinical gene panels currently used in the UK to be expanded to include *CHEK2*, *SDHA*, *SDHC* and *SDHD*. These additional CSGs also predispose to other tumour types and surveillance recommendations are available for gene carriers (35,40). In addition, for *SDHA*, *SDHC* and *SDHD*, functional investigations (e.g. *SDHB*/*SDHA* immunohistochemistry and metabolomics) are available that can aid *SDH*x variant interpretation (41,42). The associated cancer risks with *MITF* variants (observed in 10 participants) are not well defined and hence we suggest that further evidence is required before incorporating *MITF* into RCC panels.

Detection of a germline P/LP in RCC-CSG variants can enable RCC prevention strategies (e.g. renal cancer surveillance, cascade testing and awareness of non-RCC tumour risks). In some cases, it may also suggest genotype-driven therapies as exhibited with foretinib for RCC patients carrying germline *MET* variants (43). As the importance of knowledge of germline findings to determine management increases and the cost of genomic analysis falls, the indications for germline and somatic sequencing in RCC should be extended and play a large part in routine clinical care. However, the selection of genes that should be tested requires careful consideration of diagnostic yields, VUS likelihood and the clinical utility (e.g. availability of management guidelines) of diagnostic findings.

## Materials and Methods

### Participants

All subjects gave written consent; 100kGP was approved under Research Ethics Committee Ref 14/EE/1112. We studied 1336 RCC participants [64% male; mean: 61 years (±12 SD); range: 13–88 years]. Their clinical characteristics are described in Table 3. Sex reported is according to participant phenotypic sex classification at birth. Ethnicity reported is self-reported by participants and also reported based on principal component analysis performed by Genomics England (GEL). For more details, see Supplementary Methods.

Healthy unrelated parents (*n* = 5834) [mothers (*n* = 3149, mean age: 39 years) and fathers (*n* = 2685, mean age: 42 years)] of children recruited to the intellectual disorders disease group of the 100kGP rare disease domain served as a source of controls. All controls were of European ancestry and a cancer diagnosis was excluded based on available data in 100kGP. For more details on their selection, see Supplementary Methods.

### Cancer susceptibility genes

We focused on 121 CSGs previously described in the Catalogue of Somatic Mutations in Cancer (Cosmic) (44), including 18 well-established CSGs for RCC (6,45) (Supplementary Material, Table S6). Genomic positions of canonical gene transcripts were retrieved from the Ensembl database (EnsDb.Hsapiens.v86) (46) and were referenced to build GRCh38.

### Short variant analysis

Short variant (SNV and INDEL) analysis was based on variants extracted for the 1336 RCC participants from the germline aggregate multi-sample VCF (aggV2) available in the 100kGP Main Programme V10 data release. In summary, extracted variants were annotated using the variant effect predictor (VEP) (v99) (47) and additional filtering was applied to include only rare variants (maximum minor allele frequency across all populations in the Genome Aggregation Database <0.5%) (48) in our selected 121 CSGs. We then prioritized the variants to assess their potential clinical relevance. Firstly, we selected those with a ‘HIGH’ impact VEP severity rating, which includes all LoF variants: stop-gained, frameshift or splice-site disruption. Secondly, we sought out functionally important missense variants and inframe indels. These variants have a ‘MODERATE’ impact rating and missense variants were only included if they were predicted to be deleterious by SIFT (49), possibly/probably damaging by Polyphen (50) and had a CADD Phred (51) score ≥20; inframe indels were included if they had a CADD Phred score ≥20. The variants were classified based on ACMG/Association for Molecular Pathology criteria (52) and, after further manual curation, were assigned to five distinct categories; P, LP, VUS, likely benign or benign. VUSs were further subclassified to hot/warm/tepid or cool/cold/ice cold VUS according to the ACGS guidelines (16). For more details on the filtering and classification, see Supplementary Methods. The bioinformatics workflow is visualized in Supplementary Material, Figure S2.

### SV analysis

SV analysis was based on the 100kGP Main Programme V8 data release. Individual VCFs with SV calls were available for 1254 of 1336 RCC participants. Germline SVs were interrogated using an adapted version of the PCAWG-SV-merge pipeline (53). For more detailed methodology, see Supplementary Methods.

### Mutational signatures in tumours

Mutational signatures were computed by 100kGP using NNLS R package (54) based on Cosmic version 2 cancer signatures (44). We examined the mutational signatures present in matched somatic RCC samples of RCC participants with germline variants in a MMR gene (*MLH1*, *MSH2*, *MSH6* and *PMS2*) or *POLE*. The presence of signatures 6, 15, 20 or 26 was interpreted as indicative of MMR deficiency and the presence of signature 10 was interpreted as POLE exonuclease deficiency.

### Statistical analysis

The relationship between age of RCC onset and germline P/LP variant status was evaluated using Welch’s two-sample *t*-test. Fisher’s exact test was applied for differences in histology, stage and sex between participants with and without P/LP variants. For the participants whose histology or stage was not available, these cases were excluded from the statistical analysis. For participants with multiple tumours, analysis of histology and stage was based on the first diagnosed tumour. The Burden test analysis was performed using Fisher’s exact test on carrier count of short variants which passed our filters aggregated per gene in RCC participants of European ancestry compared with controls of European ancestry. Multiple testing correction was performed by FDR. Statistical analyses were performed using R studio (v3.4.4) (55). A two-sided *P*-value of <0.05 was considered to be statistically significant.

## Data availability

The WGS data analyzed in this study can be accessed through a secure research environment hosted within the GEL Data Centre https://www.genomicsengland.co.uk/about-gecip/for-gecip-members/data-and-data-access/. In order to gain access, researchers will need to apply and become a member of the Genomics England Clinical Interpretation Partnerships (GeCIPs). Researchers, clinicians and students can apply to join any GeCIP domain that is relevant to their intended research projects. https://www.genomicsengland.co.uk/about-gecip/joining-research-community/.

*Conflict of Interest statement.* S.T. has received speaking fees from Roche, Astra Zeneca, Novartis and Ipsen. S.T. has the following patents filed: Indel mutations as a therapeutic target and predictive biomarker PCTGB2018/051892 and PCTGB2018/051893 and ccRCC Biomarkers P113326GB. E.R.M. has received speaker fees from MSD.

## Supplementary Material

HMG_Supplementary_Tables_1_2_5_6_revised_ddac089

Supplementary_Material_ddac089

## References

[1] Siegel, R.L., Miller, K.D. and Jemal, A. (2018) Cancer statistics. CA. Cancer J. Clin., 68, 7–30. 29313949 10.3322/caac.21442

[2] Capitanio, U., Bensalah, K., Bex, A., Boorjian, S.A., Bray, F., Coleman, J., Gore, J.L., Sun, M., Wood, C. and Russo, P. (2019) Epidemiology of renal cell carcinoma. Eur. Urol., 75, 74–84. 30243799 10.1016/j.eururo.2018.08.036 PMC8397918

[3] Moch, H., Cubilla, A.L., Humphrey, P.A., Reuter, V.E. and Ulbright, T.M. (2016) The 2016 WHO classification of tumours of the urinary system and male genital organs-part A: renal, penile, and resticular tumours. Eur. Urol., 70, 93–105. 26935559 10.1016/j.eururo.2016.02.029

[4] Linehan, W.M., Walther, M.M. and Zbar, B. (2003) The genetic basis of cancer of the kidney. J. Urol., 170, 2163–2172. 14634372 10.1097/01.ju.0000096060.92397.ed

[5] Menko, F.H. and Maher, E.R. (2016) Diagnosis and management of hereditary renal cell cancer. Recent Results Cancer Res., 205, 85–104. 27075350 10.1007/978-3-319-29998-3_6

[6] Carlo, M.I., Hakimi, A.A., Stewart, G.D., Bratslavsky, G., Brugarolas, J., Chen, Y.B., Linehan, W.M., Maher, E.R., Merino, M.J., Offit, K. et al. (2019) Familial kidney cancer: implications of new syndromes and molecular insights. Eur. Urol., 76, 754–764. 31326218 10.1016/j.eururo.2019.06.015 PMC7673107

[7] Purdue, M.P., Song, L., Scelo, G., Houlston, R.S., Wu, X., Sakoda, L.C., Thai, K., Graff, R.E., Rothman, N., Brennan, P. et al. (2020) Pathway analysis of renal cell carcinoma genome-wide association studies identifies novel associations. Cancer Epidemiol. Biomark. Prev., 29, 2065–2069. 10.1158/1055-9965.EPI-20-0472 PMC9438507 32732251

[8] Scelo, G., Purdue, M.P., Brown, K.M., Johansson, M., Wang, Z., Eckel-Passow, J.E., Ye, Y., Hofmann, J.N., Choi, J., Foll, M. et al. (2017) Genome-wide association study identifies multiple risk loci for renal cell carcinoma. Nat. Commun., 8, 15724. 28598434 10.1038/ncomms15724 PMC5472706

[9] Carlo, M.I., Mukherjee, S., Mandelker, D., Vijai, J., Kemel, Y., Zhang, L., Knezevic, A., Patil, S., Ceyhan-Birsoy, O., Huang, K.C. et al. (2018) Prevalence of germline mutations in cancer susceptibility genes in patients with advanced renal cell carcinoma. JAMA Oncol., 4, 1228–1235. 29978187 10.1001/jamaoncol.2018.1986 PMC6584283

[10] Wu, J., Wang, H., Ricketts, C.J., Yang, Y., Merino, M.J., Zhang, H., Shi, G., Gan, H., Linehan, W.M., Zhu, Y. et al. (2019) Germline mutations of renal cancer predisposition genes and clinical relevance in Chinese patients with sporadic, early-onset disease. Cancer, 125, 1060–1069. 30548481 10.1002/cncr.31908 PMC8201926

[11] Abou Alaiwi, S., Nassar, A.H., Adib, E., Groha, S.M., Akl, E.W., McGregor, B.A., Esplin, E.D., Yang, S., Hatchell, K., Fusaro, V. et al. (2021) Trans-ethnic variation in germline variants of patients with renal cell carcinoma. Cell Rep., 34, 108926. 33789101 10.1016/j.celrep.2021.108926

[12] Nguyen, K.A., Syed, J.S., Espenschied, C.R., LaDuca, H., Bhagat, A.M., Suarez-Sarmiento, A., O'Rourke, T.K., Jr., Brierley, K.L., Hofstatter, E.W. and Shuch, B. (2017) Advances in the diagnosis of hereditary kidney cancer: Initial results of a multigene panel test. Cancer, 123, 4363–4371. 28787086 10.1002/cncr.30893

[13] Caulfield, M., Davies, J., Dennys, M., Elbahy, L., Fowler, T., Hill, S., Hubbard, T., Jostins, L., Maltby, N. and Mahon-Pearson, J. (2017) The National Genomics Research and Healthcare Knowledgebase. Genomics England, UK. 10.6084/m9.figshare.4530893.v3.

[14] Whitworth, J., Smith, P.S., Martin, J.E., West, H., Luchetti, A., Rodger, F., Clark, G., Carss, K., Stephens, J., Stirrups, K. et al. (2018) Comprehensive cancer-predisposition gene testing in an adult multiple primary tumor series shows a broad range of deleterious variants and atypical tumor phenotypes. Am. J. Hum. Genet., 103, 3–18. 29909963 10.1016/j.ajhg.2018.04.013 PMC6037202

[15] Tavtigian, S.V., Greenblatt, M.S., Harrison, S.M., Nussbaum, R.L., Prabhu, S.A., Boucher, K.M., Biesecker, L.G. and ClinGen Sequence Variant Interpretation Working Group (ClinGen SVI) (2018) Modeling the ACMG/AMP variant classification guidelines as a Bayesian classification framework. Genet. Med., 20, 1054–1060. 29300386 10.1038/gim.2017.210 PMC6336098

[16] Sian Ellard, E.L.B., Owens, M., Eccles, D.M., Turnbull, C., Stephen, A., Scott, R., Deans, Z.C., Lester, T., Campbell, J., Newman, W.G. et al. (2018) ACGS Best Practice Guidelines for Variant Classification 2018. Association for Clinical Genomic Science, UK, https://www.acgs.uk.com/media/11631/uk-practice-guidelines-for-variant-classification-v4-01-2020.pdf.

[17] Smith, P.S., West, H., Whitworth, J., Castle, B., Sansbury, F.H., Warren, A.Y., Woodward, E.R., Tischkowitz, M. and Maher, E.R. (2021) Pathogenic germline variants in patients with features of hereditary renal cell carcinoma: Evidence for further locus heterogeneity. Genes Chromosomes Cancer, 60, 5–16. 32830346 10.1002/gcc.22893

[18] Margulis, V., Lin, J., Yang, H., Wang, W., Wood, C.G. and Wu, X. (2008) Genetic susceptibility to renal cell carcinoma: the role of DNA double-strand break repair pathway. Cancer Epidemiol. Biomark. Prev., 17, 2366–2373. 10.1158/1055-9965.EPI-08-0259 PMC2581835 18768505

[19] Huang, K.L., Mashl, R.J., Wu, Y., Ritter, D.I., Wang, J., Oh, C., Paczkowska, M., Reynolds, S., Wyczalkowski, M.A., Oak, N. et al. (2018) Pathogenic germline variants in 10,389 adult cancers. Cell, 173(355–370), e314. 10.1016/j.cell.2018.03.039 PMC5949147 29625052

[20] National Genomic Test Directory for Rare and Inherited Disease: Inherited Renal Cancer. National Health Service National Genomic Test Directory. National Health Service, England. URL: https://www.england.nhs.uk/publication/national-genomic-test-directories/.

[21] Easton, D.F., Pharoah, P.D., Antoniou, A.C., Tischkowitz, M., Tavtigian, S.V., Nathanson, K.L., Devilee, P., Meindl, A., Couch, F.J., Southey, M. et al. (2015) Gene-panel sequencing and the prediction of breast-cancer risk. N. Engl. J. Med., 372, 2243–2257. 26014596 10.1056/NEJMsr1501341 PMC4610139

[22] Cybulski, C., Wokolorczyk, D., Kladny, J., Kurzawski, G., Suchy, J., Grabowska, E., Gronwald, J., Huzarski, T., Byrski, T., Gorski, B. et al. (2007) Germline CHEK2 mutations and colorectal cancer risk: different effects of a missense and truncating mutations? Eur. J. Hum. Genet., 15, 237–241. 17106448 10.1038/sj.ejhg.5201734

[23] Cybulski, C., Wokolorczyk, D., Huzarski, T., Byrski, T., Gronwald, J., Gorski, B., Debniak, T., Masojc, B., Jakubowska, A., Gliniewicz, B. et al. (2006) A large germline deletion in the Chek2 kinase gene is associated with an increased risk of prostate cancer. J. Med. Genet., 43, 863–866. 17085682 10.1136/jmg.2006.044974 PMC2563179

[24] Cybulski, C., Gorski, B., Huzarski, T., Masojc, B., Mierzejewski, M., Debniak, T., Teodorczyk, U., Byrski, T., Gronwald, J., Matyjasik, J. et al. (2004) CHEK2 is a multiorgan cancer susceptibility gene. Am. J. Hum. Genet., 75, 1131–1135. 15492928 10.1086/426403 PMC1182149

[25] Ge, Y., Wang, Y., Shao, W., Jin, J., Du, M., Ma, G., Chu, H., Wang, M. and Zhang, Z. (2016) Rare variants in BRCA2 and CHEK2 are associated with the risk of urinary tract cancers. Sci. Rep., 6, 33542. 27632928 10.1038/srep33542 PMC5025839

[26] Zlowocka-Perlowska, E., Narod, S.A. and Cybulski, C. (2019) CHEK2 alleles predispose to renal cancer in Poland. JAMA Oncol., 5, 576. 10.1001/jamaoncol.2019.0022 30816943

[27] Bertolotto, C., Lesueur, F., Giuliano, S., Strub, T., de Lichy, M., Bille, K., Dessen, P., d'Hayer, B., Mohamdi, H., Remenieras, A. et al. (2011) A SUMOylation-defective MITF germline mutation predisposes to melanoma and renal carcinoma. Nature, 480, 94–98. 22012259 10.1038/nature10539

[28] Yokoyama, S., Woods, S.L., Boyle, G.M., Aoude, L.G., MacGregor, S., Zismann, V., Gartside, M., Cust, A.E., Haq, R., Harland, M. et al. (2011) A novel recurrent mutation in MITF predisposes to familial and sporadic melanoma. Nature, 480, 99–103. 22080950 10.1038/nature10630 PMC3266855

[29] Paillerets, B.B., Lesueur, F. and Bertolotto, C. (2014) A germline oncogenic MITF mutation and tumor susceptibility. Eur. J. Cell Biol., 93, 71–75. 24290354 10.1016/j.ejcb.2013.10.002

[30] Guhan, S.M., Artomov, M., McCormick, S., Njauw, C., Stratigos, A.J., Shannon, K., Ellisen, L.W. and Tsao, H. (2020) Cancer risks associated with the germline MITF(E318K) variant. Sci. Rep., 10, 17051. 33051548 10.1038/s41598-020-74237-z PMC7555480

[31] Ricketts, C.J., Forman, J.R., Rattenberry, E., Bradshaw, N., Lalloo, F., Izatt, L., Cole, T.R., Armstrong, R., Kumar, V.K., Morrison, P.J. et al. (2010) Tumor risks and genotype-phenotype-proteotype analysis in 358 patients with germline mutations in SDHB and SDHD. Hum. Mutat., 31, 41–51. 19802898 10.1002/humu.21136

[32] Ricketts, C., Woodward, E.R., Killick, P., Morris, M.R., Astuti, D., Latif, F. and Maher, E.R. (2008) Germline SDHB mutations and familial renal cell carcinoma. J. Natl. Cancer Inst., 100, 1260–1262. 18728283 10.1093/jnci/djn254

[33] Casey, R.T., Warren, A.Y., Martin, J.E., Challis, B.G., Rattenberry, E., Whitworth, J., Andrews, K.A., Roberts, T., Clark, G.R., West, H. et al. (2017) Clinical and molecular features of renal and pheochromocytoma/paraganglioma tumor association syndrome (RAPTAS): case series and literature review. J. Clin. Endocrinol. Metab., 102, 4013–4022. 28973655 10.1210/jc.2017-00562 PMC5673270

[34] Benn, D.E., Zhu, Y., Andrews, K.A., Wilding, M., Duncan, E.L., Dwight, T., Tothill, R.W., Burgess, J., Crook, A., Gill, A.J. et al. (2018) Bayesian approach to determining penetrance of pathogenic SDH variants. J. Med. Genet., 55, 729–734. 30201732 10.1136/jmedgenet-2018-105427 PMC6252366

[35] Wong, M.Y., Andrews, K.A., Challis, B.G., Park, S.M., Acerini, C.L., Maher, E.R. and Casey, R.T. (2019) Clinical practice guidance: surveillance for phaeochromocytoma and paraganglioma in paediatric succinate dehydrogenase gene mutation carriers. Clin. Endocrinol., 90, 499–505. 10.1111/cen.13926 PMC6850004 30589099

[36] Shuch, B., Vourganti, S., Ricketts, C.J., Middleton, L., Peterson, J., Merino, M.J., Metwalli, A.R., Srinivasan, R. and Linehan, W.M. (2014) Defining early-onset kidney cancer: implications for germline and somatic mutation testing and clinical management. J. Clin. Oncol., 32, 431–437. 24378414 10.1200/JCO.2013.50.8192 PMC3912328

[37] Reaume, M.N., Graham, G.E., Tomiak, E., Kamel-Reid, S., Jewett, M.A., Bjarnason, G.A., Blais, N., Care, M., Drachenberg, D., Gedye, C. et al. (2013) Canadian guideline on genetic screening for hereditary renal cell cancers. Can. Urol. Assoc. J., 7, 319–323. 24319509 10.5489/cuaj.1496 PMC3854468

[38] Bratslavsky, G., Mendhiratta, N., Daneshvar, M., Brugarolas, J., Ball, M.W., Metwalli, A., Nathanson, K.L., Pierorazio, P.M., Boris, R.S., Singer, E.A. et al. (2021) Genetic risk assessment for hereditary renal cell carcinoma: clinical consensus statement. Cancer, 127, 3957–3966. 34343338 10.1002/cncr.33679 PMC8711633

[39] Hampel, H., Bennett, R.L., Buchanan, A., Pearlman, R., Wiesner, G.L., Guideline Development Group, A.C.O.M.G., Genomics Professional, P, Guidelines, C and National Society of Genetic Counselors Practice Guidelines, C (2015) A practice guideline from the American College of Medical Genetics and Genomics and the National Society of Genetic Counselors: referral indications for cancer predisposition assessment. Genet. Med., 17, 70–87. 25394175 10.1038/gim.2014.147

[40] Taylor, A., Brady, A.F., Frayling, I.M., Hanson, H., Tischkowitz, M., Turnbull, C., Side, L. and Group, U.K.C.G (2018) Consensus for genes to be included on cancer panel tests offered by UK genetics services: guidelines of the UK Cancer Genetics Group. J. Med. Genet., 55, 372–377. 29661970 10.1136/jmedgenet-2017-105188 PMC5992364

[41] van Nederveen, F.H., Gaal, J., Favier, J., Korpershoek, E., Oldenburg, R.A., de Bruyn, E.M., Sleddens, H.F., Derkx, P., Riviere, J., Dannenberg, H. et al. (2009) An immunohistochemical procedure to detect patients with paraganglioma and phaeochromocytoma with germline SDHB, SDHC, or SDHD gene mutations: a retrospective and prospective analysis. Lancet Oncol., 10, 764–771. 19576851 10.1016/S1470-2045(09)70164-0 PMC4718191

[42] Kim, E., Wright, M.J., Sioson, L., Novos, T., Gill, A.J., Benn, D.E., White, C., Dwight, T. and Clifton-Bligh, R.J. (2017) Utility of the succinate: Fumarate ratio for assessing SDH dysfunction in different tumor types. Mol. Genet. Metab. Rep., 10, 45–49. 28070496 10.1016/j.ymgmr.2016.12.006 PMC5219629

[43] Choueiri, T.K., Vaishampayan, U., Rosenberg, J.E., Logan, T.F., Harzstark, A.L., Bukowski, R.M., Rini, B.I., Srinivas, S., Stein, M.N., Adams, L.M. et al. (2013) Phase II and biomarker study of the dual MET/VEGFR2 inhibitor foretinib in patients with papillary renal cell carcinoma. J. Clin. Oncol., 31, 181–186. 23213094 10.1200/JCO.2012.43.3383 PMC3532390

[44] Tate, J.G., Bamford, S., Jubb, H.C., Sondka, Z., Beare, D.M., Bindal, N., Boutselakis, H., Cole, C.G., Creatore, C., Dawson, E. et al. (2019) COSMIC: the catalogue of somatic mutations in cancer. Nucleic Acids Res., 47, D941–D947. 30371878 10.1093/nar/gky1015 PMC6323903

[45] Maher, E.R. (2018) Hereditary renal cell carcinoma syndromes: diagnosis, surveillance and management. World J. Urol., 36, 1891–1898. 29680948 10.1007/s00345-018-2288-5 PMC6280834

[46] Howe, K.L., Achuthan, P., Allen, J., Allen, J., Alvarez-Jarreta, J., Amode, M.R., Armean, I.M., Azov, A.G., Bennett, R., Bhai, J. et al. (2021) Ensembl 2021. Nucleic Acids Res., 49, D884–D891. 33137190 10.1093/nar/gkaa942 PMC7778975

[47] McLaren, W., Gil, L., Hunt, S.E., Riat, H.S., Ritchie, G.R., Thormann, A., Flicek, P. and Cunningham, F. (2016) The ensembl variant effect predictor. Genome Biol., 17, 122. 27268795 10.1186/s13059-016-0974-4 PMC4893825

[48] Karczewski, K.J., Francioli, L.C., Tiao, G., Cummings, B.B., Alfoldi, J., Wang, Q., Collins, R.L., Laricchia, K.M., Ganna, A., Birnbaum, D.P. et al. (2020) The mutational constraint spectrum quantified from variation in 141,456 humans. Nature, 581, 434–443. 32461654 10.1038/s41586-020-2308-7 PMC7334197

[49] Sim, N.L., Kumar, P., Hu, J., Henikoff, S., Schneider, G. and Ng, P.C. (2012) SIFT web server: predicting effects of amino acid substitutions on proteins. Nucleic Acids Res., 40, W452–W457. 22689647 10.1093/nar/gks539 PMC3394338

[50] Adzhubei, I., Jordan, D.M. and Sunyaev, S.R. (2013) Predicting functional effect of human missense mutations using PolyPhen-2. Curr Protoc Hum Genet, Chapter, 7(Unit7), 20. 10.1002/0471142905.hg0720s76 PMC4480630 23315928

[51] Kircher, M., Witten, D.M., Jain, P., O'Roak, B.J., Cooper, G.M. and Shendure, J. (2014) A general framework for estimating the relative pathogenicity of human genetic variants. Nat. Genet., 46, 310–315. 24487276 10.1038/ng.2892 PMC3992975

[52] Richards, S., Aziz, N., Bale, S., Bick, D., Das, S., Gastier-Foster, J., Grody, W.W., Hegde, M., Lyon, E., Spector, E. et al. (2015) Standards and guidelines for the interpretation of sequence variants: a joint consensus recommendation of the American College of Medical Genetics and Genomics and the Association for Molecular Pathology. Genet. Med., 17, 405–424. 25741868 10.1038/gim.2015.30 PMC4544753

[53] Consortium, I.T.P.-C.A.o.W.G (2020) Pan-cancer analysis of whole genomes. Nature, 578, 82–93. 32025007 10.1038/s41586-020-1969-6 PMC7025898

[54] Muller, M. and Von Stokkum, M.H.I. (2015) NNLS Package: The Lawson-Hanson Algorithm for Non-Negative Least Squares (NNLS), 1.4 edn. The comprehensive R archive Network (CRAN), Online. https://cran.r-project.org/web/packages/nnls/nnls.pdf.

[55] RStudio Team (2020) RStudio: Integrated Development for R. RStudio, PBC, Boston, MA, http://www.rstudio.com/.

[56] Yin, T., Cook, D. and Lawrence, M. (2012) ggbio: an R package for extending the grammar of graphics for genomic data. Genome Biol., 13, R77. 22937822 10.1186/gb-2012-13-8-r77 PMC4053745

[57] Jay, J.J. and Brouwer, C. (2016) Lollipops in the clinic: information dense mutation plots for precision medicine. PLoS One, 11, e0160519. 10.1371/journal.pone.0160519 PMC4973895 27490490

