# Supplementary material for: Frequency of pathogenic germline variants in cancer susceptibility genes in 1336 renal cell carcinoma cases
Source: Hum Mol Genet. 2022 Apr 20;31(17):3001–11. doi: 10.1093/hmg/ddac089 (PMC9433729; doi:10.1093/hmg/ddac089)
Supplement: Supplementary_Material_ddac089 [file supplementary_material_ddac089.docx]

## **Supplementary Material**

## **Frequency of pathogenic germline variants in cancer susceptibility genes in 1,336 renal cell carcinoma cases.**

## Bryndis Yngvadottir^1,#^, Avgi Andreou^1, #^, Laia Bassaganyas^1^, Alexey Larionov^1,2^, Alex J Cornish^3^, Daniel Chubb^3^, Charlie N Saunders^3^, Philip Smith^1^, Huairen Zhang^1^, Yasemin Cole^1^, Genomics England Research Consortium, James Larkin^4^, Lisa Browning^5,6^, Samra Turajlic^7^, Kevin Litchfield^8^, Richard S Houlston^3^, Eamonn R Maher^1,^*

## # These authors contributed equally to this work

* Corresponding Author

**Affiliations:**

## Department of Medical Genetics, School of Clinical Medicine, University of Cambridge, Cambridge, CB2 0QQ, UK

1. SWEE, Cranfield University, Cranfield, UK
2. Division of Genetics and Epidemiology, The Institute of Cancer Research, London, SW7 3RP, UK.

Department of Medical Oncology, Royal Marsden NHS Foundation Trust, London, UK.

Department of Cellular Pathology, Oxford University Hospitals NHS Foundation Trust, John Radcliffe Hospital, Oxford, UK OX3 9DU

NIHR Oxford Biomedical Research Centre, Oxford University Hospitals NHS Foundation Trust, Oxford, UK.

1. Cancer Dynamics Laboratory, The Francis Crick Institute, London and Renal and Skin Units, The Royal Marsden NHS Foundation Trust, London, UK.
2. University College London Cancer Institute, Paul O'Gorman Building, London, UK.

**Corresponding Author:** Professor Eamonn R Maher, Department of Medical Genetics, University of Cambridge, Box 238, Cambridge Biomedical Campus, Cambridge, CB2 0QQ, UK, Tel +44 01223 746715, Fax +44 01223 746777, Email: [erm1000@medschl.cam.ac.uk](mailto:erm1000@medschl.cam.ac.uk)

# Supplementary Methods

## Participants

The 100K Genomes Project (100KGP) is a national genome sequencing initiative approved by the Health Research Authority Research Ethics Committee (REC Ref 14/EE/1112) and is managed by Genomics England (GEL) (1). The 1,336 RCC cases included in this study were selected by reviewing clinical data of participants recruited under the Renal Cancer disease group in the Cancer domain of the 100KGP. This data was provided in Labkey tables in GEL’s Research Environment and includes information provided by the recruiting centres and the National Cancer Registration and Analysis Service (NCRAS). To define our RCC cohort, we excluded RCC participants reported as having a non-RCC histology, defined by the following histology descriptions: "transitional cell carcinoma", "urothelial", "nephroblastoma", "pseudosarcomatous", "carcinoid", "acquired cystic disease", "Ewing's sarcoma", "lymphoma", "squamous cell carcinoma" and "neuroendocrine".

Sex reported is according to participant phenotypic sex classification at birth. Ethnicity reported is self-reported by participants as per the UK 2001 Census ethnicity categories (2). Ethnicity is also reported based on principal components analysis (PCA) performed by Genomics England (GEL) using a Random Forest ancestry model fit (3). The broad genetic ancestry was estimated using ethnicities from the 1000 genomes project phase 3 (1KGP3) (4), by generating principle components (PCs) for 1KGP3 samples and projecting all aggV2 participants onto these. The five broad super-populations defined were: African, Admixed American, East Asian, South Asian and European. The ethnicity of RCC participants reported in **Table** 3 is as assigned with a probability of ≥0.9 for any one ancestry of the five broad super-populations defined. Participant numbers with South Asian and East Asian ancestries were combined in **Table 3**. “Other” ancestry in **Table 3** refers to those participants with ancestry assigned with a probability <0.9 for any one ancestry.

Controls

Healthy unrelated parents (n=5,834) (mothers and fathers) of children recruited to the intellectual disorders disease group of the 100KGP rare disease domain labelled with affection status “unaffected” in the Labkey “rare disease pedigree” table served as a source of controls. This identified 6,214 potential controls. To ensure that controls did not have a known cancer diagnosis we excluded any parents whose participant id was linked to a cancer related HPO term in the Labkey “rare disease participant phenotype” table (n=2) or a cancer diagnosis provided in the “NHS cancer registry” Labkey table (n=378). This resulted in excluding 380 controls from the initial 6,214 control cohort identified, leaving 5,834 “cancer-free” controls [mothers (n=3,149, mean age 39 years) and fathers (n=2,685, mean age 42 years]. All controls were of European ancestry.

## Whole Genome Sequencing

Whole genome 150bp paired-end TruSeq PCR–free libraries were sequenced on a single lane using Illumina (San Diego, USA) HiSeq X technology and uniformly processed on the Illumina North Star Version 4 Whole Genome Sequencing Workflow (NSV4, version 2.6.53.23). Raw sequencing data was aligned to the NCBI GRCh38 assembly (with decoys) using iSAAC Aligner (version 03.16.02.19) and small germline variants were called using Starling (version 2.4.7).

## Short variant analysis

Short variant analysis (SNV and INDEL) was based on variants extracted for the 1,336 RCC participants from the germline multi-sample VCF (aggV2) available in the 100KGP Main Programme V10 data release. The multi-sample VCF was produced by GEL’s bioinformatics team as described below. The variant calls were performed with Illumina’s gVCF genotyper (version: 2019.02.26) and initially included all 78,195 participants from both Cancer and Rare Disease Domains that passed the following quality control filters: sample contamination < 0.03, ratio of SNV heterozygous to homozygous calls < 3, total number of SNVs between 3.2M and 4.7M, array concordance > 90%, median fragment size > 250bp, excess of chimeric reads < 5%, percentage of mapped reads > 60% and percentage AT dropout < 10%. The VCF filter was set to ‘PASS’ based on the following parameters: missingness (fully missing genotypes with DP=0) ≤ 5%, coverage (Median Depth) ≥ 10X, GQ (Median GQ) ≥ 15, ABratio (Percentage of het calls not showing significant allele imbalance for reads supporting the ref and alt alleles) ≥ 25%, completeGTRatio (Percentage of complete sites (sites with no missing data)) ≥ 50%, phwe_eur (p-value for deviations from HWE in unrelated samples of inferred European ancestry) ≥ 1e-5.

Using the above multi-sample VCF file, we extracted data for 1,336 RCC cases and 5,834 healthy controls. We annotated the extracted variants using VEP (v99) (5) and applied the additional filtering: QUAL≥30, HWE ≥0.001 and gnomAD AF (18) < 0.5% to include only rare variants. We selected the canonical transcript for each CSG and focused our analysis on variants with a "HIGH" and a "MODERATE" impact VEP severity rating. We included all "HIGH" impact variants which are loss of function variants (stop-gained, frameshift or splice-site disruption variants). Functionally important missense variants defined as those predicted by SIFT (6) to be deleterious and by Polyphen (7) to be “possibly/probably damaging” and have a CADD Phred (8) score ≥20 and inframe indels with a CADD Phred score ≥20 were also retained. Variants were further filtered based on their pathogenic interpretation and review status on ClinVar (9), with variants annotated as: "pathogenic/likely pathogenic/risk factor" and having a good review status defined as: "reviewed by experts/multiple submitters/no conflicts/single submitter" retained. The ACMG/AMP classification (10) of variants was further assessed using InterVar (11). Finally, manual curation was performed incorporating the above criteria and literature review to classify variants as pathogenic (P), likely pathogenic (LP), variants of uncertain significance (VUS), benign (B) or likely benign (LB).

The quantitative Bayesian framework provided by Tavtigian et al (2018) (12) was used to calculate a posterior probability for pathogenicity and VUSs were then subclassified to hot/warm/tepid or cool/cold/ice cold VUS according to the ACGS guidelines (13).

Whether any of the reported variants were located in a hotspot was determined by combining evidence from Cosmic (14), InterVar (11) and 2 other hotspot databases (<https://www.cancerhotspots.org/> (15, 16) and <https://www.3dhotspots.org/> (17)).

## Candidate rare structural variants analysis

SV analysis was based on the 100KGP Main Programme V8 data release. Individual VCFs with structural variant calls were available for 1,254 of our 1,336 RCC cases. Germline SVs, including deletions, duplications, inversions and translocations, were interrogated using an adapted version of the PCAWG-SV-merge pipeline (18). Consensus SV breakpoint calling was based on Delly (19), Manta (20) and Lumpy (21). SVs involving the 121 CSGs were annotated with AnnotSV version 2.1 (22) applying the following filters: (i) only variants predicted by all three callers were retained; (ii) each SV breakpoint had to be covered by ≥15 reads by callers; (iii) at least one breakpoint had to fall within the gene boundaries (i.e. gene-disrupting variants); (iv) a filter of maximum allele frequency (MAX AF) ≤ 5% (highest allele frequency observed in any population from the 1,000 Genomes Project (4) was set to include only rare variants. All variants were manually confirmed using the Integrative Genomics Viewer (IGV) (23).

To evaluate SVs most likely to have a causative functional impact in RCC, we selected a subset of ‘high-priority germline SVs’, focusing on alterations in six RCC-CSGs (*VHL*, *CHEK2*, *PBRM1*, *PTEN*, *MAX* and *SDHB*) and other CSGs (*NF1* and *RB1*). This retained seven deletions (**Supplementary Table 2**) affecting 24 participants, but only two deletions (1 in *VHL* and 1 in *CHEK2,* **Figure 2**) were considered to be pathogenic without additional functional validation, highlighting the need for a comprehensive screening of genes beyond the characterisation of SNVs.

The one SV affecting the *VHL* gene was a 13kb deletion that removes 5kb of the gene. Due to the highly probable pathogenicity of the aberration, we searched for other participants potentially carrying the same/similar event by relaxing the quality filtering parameter of ‘≥15 reads in 3 SV callers’ to ‘≥15 reads in ≥2 SV callers’. This allowed us to identify another individual harbouring a similar deletion (Participant B, **Figure 2**) presenting with a VHL disease phenotype without carrying any other P/LP SNVs or INDELs. In this case, there is a 10kb deletion starting 3kb upstream of the *VHL* non-coding sequence which includes a 5kb deletion of the gene sequence, with the second breakpoint falling only 62bp away from the second breakpoint of the deletion in Participant A.

The most recurrent rare structural variant (SV) was a ~900-bp deletion falling within an intronic region of the *PTEN* (RCC-CSG) (15/1254 [1.2%]), which had already been observed with a maximum allele frequency of 2.9% across populations from the 1,000 Genomes Project (4). There was also a recurrent 1.3Mb inversion (10/1254 [0.8%]) in the *SDHA* (RCC-CSG). For this inversion, it should be noted that while each of the first breakpoints map to introns within the gene the second breakpoints map further downstream of the *SDHA* within the *SDHAP3* pseudogene indicating the possibility of a false positive mapping issue. We also detected a previously unknown ~600-bp duplication within an intronic region in the *EZH2* (other CSGs) (10/1254 [0.8%]). The remaining rearrangements were detected in <75 individuals (<0.6%), indicating that germline truncating SVs may be rare events among the RCC participants. On the other hand, most CSGs were affected by a unique alteration, with the exception of the *SDHA* gene, in which we identified a ~370-kb duplication (n=1) and a 1.3Mb inversion (n=103), and the *EZH2* gene, found altered by the previously mentioned duplication and by a ~3.7-kb deletion (n=1) (**Supplementary Table 2**).

Exceptionally, one individual harboured two simultaneous SV alterations; a ~200-kb duplication and a ~51-kb head-to-head inversion, in the *ERCC3* (other CSGs) (**Supplementary Figure 3**). This pattern of complex structural aberrations, so-called ‘fold-back inversions’ or ‘inverted duplications’, has already been observed in other cancers both in germline and somatic genomes analysed by whole genome sequencing (WGS) (24). Interestingly, fold-back inversions were found as early events in the development of pancreatic cancer (25).

The ACMG/ClinGen CNV Loss and Gain Guidelines (2020) (26) were used to classify SV deletions and duplications deemed potentially pathogenic or VUSs of clinical relevance.

## Burden test analysis

For the burden test analysis, we included loss of function variants and functionally important missense variants and inframe indels that passed the SNV filters above in 1,183 RCC participants and 5,834 controls of European ancestry. For the burden analysis, functionally important missense variants were more strictly defined as those predicted by SIFT to be deleterious and by Polyphen to be “probably damaging” and have a CADD Phred score ≥20. Variants that passed these filters were aggregated per gene and Fisher’s exact test was applied on carrier count in European RCC participants compared to controls. Multiple testing correction was performed by False Discovery Rate (FDR).

# Supplementary References

1 Caulfield,M., Davies,J., Dennys,M., Elbahy,L., Fowler,T., Hill,S., Hubbard,T., Jostins,L., Maltby,N. and Mahon-Pearson,J. (2017) The 100,000 Genomes Project Protocol. 10.6084/m9.figshare.4530893.v3.

2 Office for National Statistics (2011): 2001 Census aggregate data (Edition: May 2011). UK Data Service. DOI: http://dx.doi.org/10.5257/census/aggregate-2001-2

3 Genomics England. Research Environemnt Documentation: *Ancestry inference*. <https://research-help.genomicsengland.co.uk/display/GERE/Ancestry+inference>.

4 Genomes Project, C., Auton, A., Brooks, L.D., Durbin, R.M., Garrison, E.P., Kang, H.M., Korbel, J.O., Marchini, J.L., McCarthy, S., McVean, G.A. *et al.* (2015) A global reference for human genetic variation. *Nature*, **526**, 68-74.

5 McLaren, W., Gil, L., Hunt, S.E., Riat, H.S., Ritchie, G.R., Thormann, A., Flicek, P. and Cunningham, F. (2016) The Ensembl Variant Effect Predictor. *Genome Biol.*, **17**, 122.

6 Sim, N.L., Kumar, P., Hu, J., Henikoff, S., Schneider, G. and Ng, P.C. (2012) SIFT web server: predicting effects of amino acid substitutions on proteins. *Nucleic Acids Res.*, **40**, W452-457.

7 Adzhubei, I., Jordan, D.M. and Sunyaev, S.R. (2013) Predicting functional effect of human missense mutations using PolyPhen-2. *Curr. Protoc. Hum. Genet.*, **Chapter 7**, Unit7 20.

8 Kircher, M., Witten, D.M., Jain, P., O'Roak, B.J., Cooper, G.M. and Shendure, J. (2014) A general framework for estimating the relative pathogenicity of human genetic variants. *Nat. Genet.*, **46**, 310-315.

9 Landrum, M.J., Lee, J.M., Benson, M., Brown, G.R., Chao, C., Chitipiralla, S., Gu, B., Hart, J., Hoffman, D., Jang, W. *et al.* (2018) ClinVar: improving access to variant interpretations and supporting evidence. *Nucleic Acids Res.*, **46**, D1062-D1067.

10 Richards, S., Aziz, N., Bale, S., Bick, D., Das, S., Gastier-Foster, J., Grody, W.W., Hegde, M., Lyon, E., Spector, E. *et al.* (2015) Standards and guidelines for the interpretation of sequence variants: a joint consensus recommendation of the American College of Medical Genetics and Genomics and the Association for Molecular Pathology. *Genet Med*, **17**, 405-424.

11 Li, Q. and Wang, K. (2017) InterVar: Clinical Interpretation of Genetic Variants by the 2015 ACMG-AMP Guidelines. *Am. J. Hum. Genet.*, **100**, 267-280.

12 Tavtigian, S.V., Greenblatt, M.S., Harrison, S.M., Nussbaum, R.L., Prabhu, S.A., Boucher, K.M., Biesecker, L.G. and ClinGen Sequence Variant Interpretation Working, G. (2018) Modeling the ACMG/AMP variant classification guidelines as a Bayesian classification framework. *Genet. Med.*, **20**, 1054-1060.

13 Sian Ellard, E.L.B., Martina Owens, Diana M Eccles, Clare Turnbull, Stephen, Abbs, Richard Scott, Zandra C Deans, Tracy Lester, Jo Campbell, William G Newman, Dominic J McMullan. (2018) ACGS Best Practice Guidelines for Variant Classification 2018. URL: <https://www.acgs.uk.com/media/11631/uk-practice-guidelines-for-variant-classification-v4-01-2020.pdf>

14 Tate, J.G., Bamford, S., Jubb, H.C., Sondka, Z., Beare, D.M., Bindal, N., Boutselakis, H., Cole, C.G., Creatore, C., Dawson, E. *et al.* (2019) COSMIC: the Catalogue Of Somatic Mutations In Cancer. *Nucleic Acids Res.*, **47**, D941-D947.

15 Chang, M.T., Asthana, S., Gao, S.P., Lee, B.H., Chapman, J.S., Kandoth, C., Gao, J., Socci, N.D., Solit, D.B., Olshen, A.B. *et al.* (2016) Identifying recurrent mutations in cancer reveals widespread lineage diversity and mutational specificity. *Nat. Biotechnol.*, **34**, 155-163.

16 Chang, M.T., Bhattarai, T.S., Schram, A.M., Bielski, C.M., Donoghue, M.T.A., Jonsson, P., Chakravarty, D., Phillips, S., Kandoth, C., Penson, A. *et al.* (2018) Accelerating Discovery of Functional Mutant Alleles in Cancer. *Cancer Discov.*, **8**, 174-183.

17 Gao, J., Chang, M.T., Johnsen, H.C., Gao, S.P., Sylvester, B.E., Sumer, S.O., Zhang, H., Solit, D.B., Taylor, B.S., Schultz, N. *et al.* (2017) 3D clusters of somatic mutations in cancer reveal numerous rare mutations as functional targets. *Genome Med.*, **9**, 4.

18 Consortium, I.T.P.-C.A.o.W.G. (2020) Pan-cancer analysis of whole genomes. *Nature*, **578**, 82-93.

19 Rausch, T., Zichner, T., Schlattl, A., Stutz, A.M., Benes, V. and Korbel, J.O. (2012) DELLY: structural variant discovery by integrated paired-end and split-read analysis. *Bioinformatics*, **28**, i333-i339.

20 Chen, X., Schulz-Trieglaff, O., Shaw, R., Barnes, B., Schlesinger, F., Kallberg, M., Cox, A.J., Kruglyak, S. and Saunders, C.T. (2016) Manta: rapid detection of structural variants and indels for germline and cancer sequencing applications. *Bioinformatics*, **32**, 1220-1222.

21 Layer, R.M., Chiang, C., Quinlan, A.R. and Hall, I.M. (2014) LUMPY: a probabilistic framework for structural variant discovery. *Genome Biol.*, **15**, R84.

22 Geoffroy, V., Herenger, Y., Kress, A., Stoetzel, C., Piton, A., Dollfus, H. and Muller, J. (2018) AnnotSV: an integrated tool for structural variations annotation. *Bioinformatics*, **34**, 3572-3574.

23 Robinson, J.T., Thorvaldsdottir, H., Winckler, W., Guttman, M., Lander, E.S., Getz, G. and Mesirov, J.P. (2011) Integrative genomics viewer. *Nat. Biotechnol.*, **29**, 24-26.

24 Hermetz, K.E., Newman, S., Conneely, K.N., Martin, C.L., Ballif, B.C., Shaffer, L.G., Cody, J.D. and Rudd, M.K. (2014) Large inverted duplications in the human genome form via a fold-back mechanism. *PLoS Genet.*, **10**, e1004139.

25 Campbell, P.J., Yachida, S., Mudie, L.J., Stephens, P.J., Pleasance, E.D., Stebbings, L.A., Morsberger, L.A., Latimer, C., McLaren, S., Lin, M.L. *et al.* (2010) The patterns and dynamics of genomic instability in metastatic pancreatic cancer. *Nature*, **467**, 1109-1113.

26 Riggs, E.R., Andersen, E.F., Cherry, A.M., Kantarci, S., Kearney, H., Patel, A., Raca, G., Ritter, D.I., South, S.T., Thorland, E.C. *et al.* (2020) Technical standards for the interpretation and reporting of constitutional copy-number variants: a joint consensus recommendation of the American College of Medical Genetics and Genomics (ACMG) and the Clinical Genome Resource (ClinGen). *Genet. Med.*, **22**, 245-257.

# Supplementary Figures

**Supplementary Figure 1**

## **:** Cancer susceptibility genes (RCC-CSGs and other CSGs (autosomal recessive and dominant)) affected by structural variants (DEL=Deletion, DUP=Duplication, INV=Inversion, TRA=Translocation).


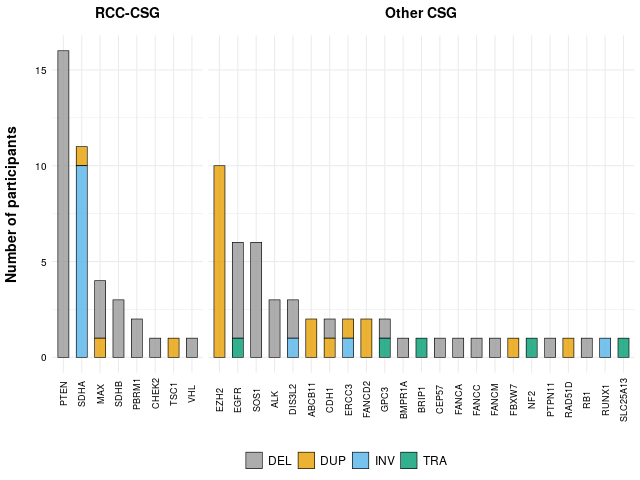


## **Supplementary Figure 2:** Bioinformatics workflow for short variants (SNVs and INDELs) and SVs.


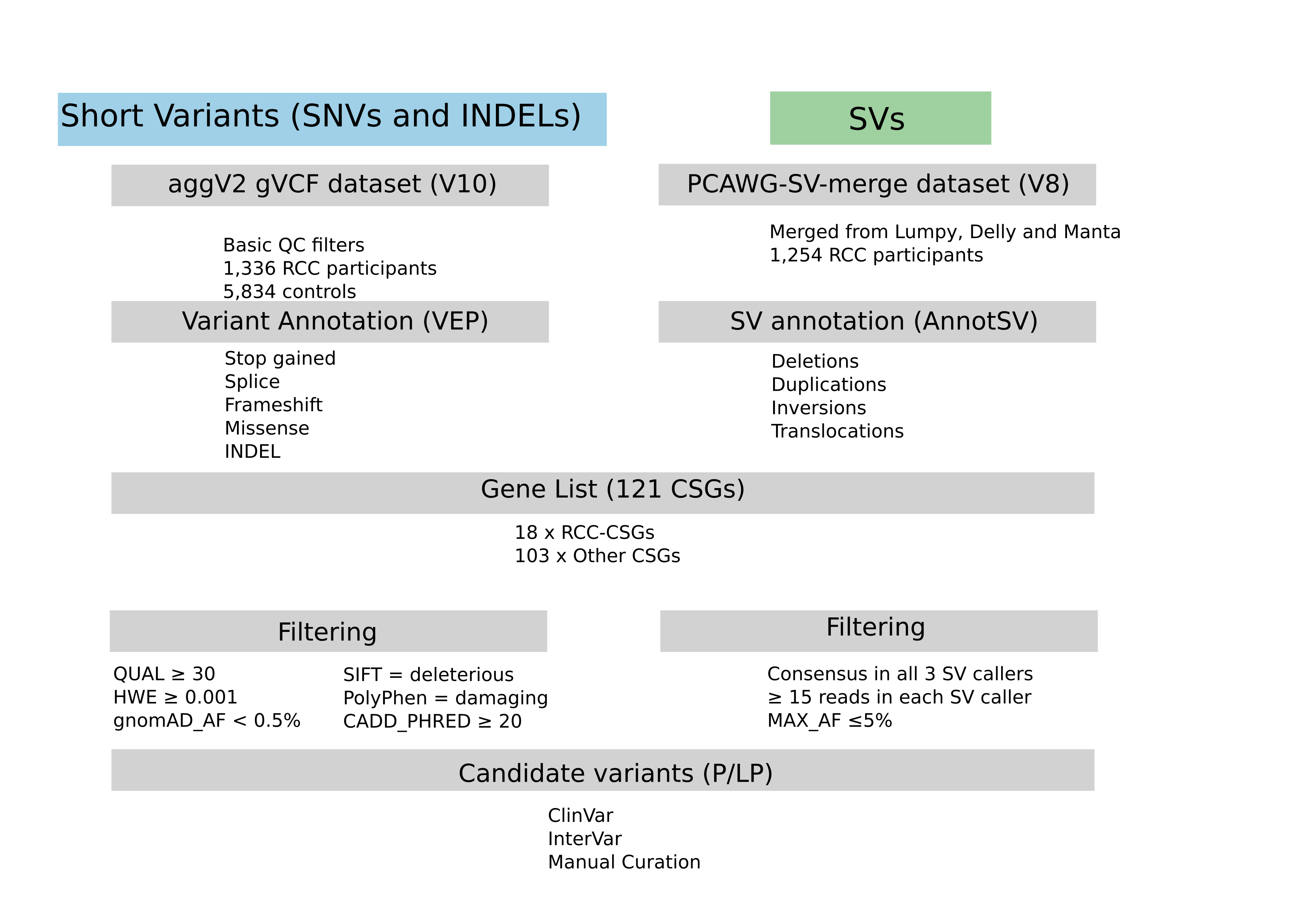


#####

#####

## **Supplementary Figure 3:** ‘Fold-back inversion’ involving the *ERCC3* gene (other CSGs). One individual harboured two simultaneous alterations, a ~200-kb duplication and a ~51-kb head-to-head inversion.


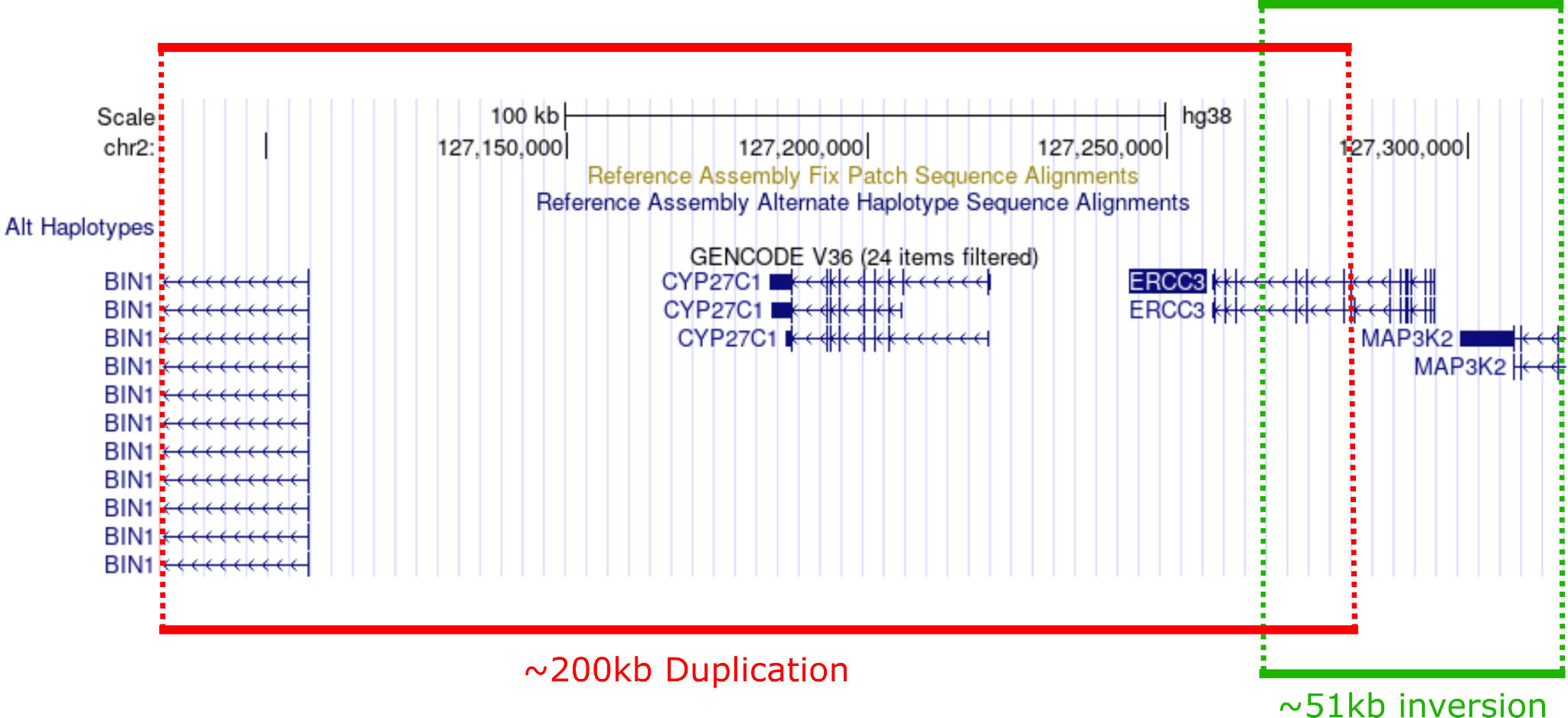


#

## **Supplementary Tables**

## **Supplementary Tables 1A, 1B, 2, 5 and 6 are provided in a separate spreadsheet included in the Supplemental Material.**

**Supplementary Table 1**: Genotype and phenotype of RCC participants with Pathogenic/Likely Pathogenic variants and clinically relevant VUSs (SNVs, indels and SVs).

**Supplementary Table 2:** Structural Variants in RCC participants.

####

#####

### **Supplementary Table 3:** RCC onset of participants with and without germline pathogenic or likely pathogenic variants.

| **Group** | **Mean (years)** | **Median (years)** | **Age range (years)** |
| --- | --- | --- | --- |
| RCC cohort  (N=1336) | 61.3 | 62 | 13-88 |
| RCC participants without P/LP variants  (n= 1251) | 61.5 | 62 | 13-88 |
| RCC participants with P/LP variants combined  (n= 85) | 58.6 | 62 | 18-84 |
| RCC participants with P/LP variants in RCC-CSG  (n=60) | 58.0 | 63 | 18-84 |
| RCC participants with P/LP variants in other CSGs  (n=27) | 59.9 | 59 | 37-80 |

## **Supplementary Table 4:** The age of RCC onset ≤40 years, ≤45 years, ≤50 years, ≤55 years and ≤60 years in RCC participants with a P/LP variant in an RCC-CSG and other CSGs.

| **RCC onset (years)** | **No of participants** | **No of participants without P/LP variant** | **No of participants with P/LP variant** | **% of participants with P/LP variant** | **No of P/LP variants (n=88)** | **No of P/LP variants in RCC-CSG (n=60)** | **No of P/LP variants in other CSG (n=28)** |
| --- | --- | --- | --- | --- | --- | --- | --- |
| **≤40** | 76 | 63 | 13 | 17.1 | 13 | 11 | 2 |
| **≤45** | 146 | 128 | 18 | 12.3 | 19 | 14 | 5 |
| **≤50** | 252 | 229 | 23 | 9.1 | 24 | 17 | 7 |
| **≤55** | 422 | 390 | 32 | 7.6 | 33 | 22 | 11 |
| **≤60** | 640 | 600 | 40 | 6.25 | 41 | 27 | 14 |

##### **Supplementary Table 5:** Fisher's exact test results carrier count per gene in European participants (cases) and controls.

## **Supplementary Table 6:** List of cancer susceptibility genes (CSGs) in alphabetical order with chromosomal coordinates (GRCh38).
